# Does More Invasive Surgery Result in Higher Patient Satisfaction? A Long-Term Follow-Up of 136 Zygomaticomaxillary Complex Fractures

**DOI:** 10.1177/19433875241286544

**Published:** 2024-10-08

**Authors:** Samin Rahbin, Ola Sunnergren, Ellen McBride, Hatef Darabi, Babak Alinasab

**Affiliations:** 1 Division of ENT Diseases, Department of Clinical Science, Intervention and Technology (CLINTEC), 27106 Karolinska Institutet and Department of ENT Diseases, Karolinska University Hospital, Stockholm, Sweden; 2 Department of Otorhinolaryngology-Head and Neck Surgery, Institute of Clinical Sciences, Sahlgrenska Academy, University of Gothenburg, Sweden; 3 Region Västra Götaland, Sahlgrenska University Hospital, Department of Otorhinolaryngology-Head and Neck Surgery, Gothenburg, Sweden; 4 25545 The Public Health Agency of Sweden, Stockholm, Sweden

**Keywords:** patient satisfaction, zygomatic fracture, zygomaticomaxillary complex, facial asymmetry, surgical wound infection, antibiotic, prophylaxis

## Abstract

**Study Design:**

Retrospective with follow-up.

**Objective:**

To evaluate the long-term satisfaction of surgically treated patients with zygomaticomaxillary complex (ZMC) fractures in relation to the use of internal fixation, number of fixation points, and orbital floor (OF) reconstruction. Secondary objectives were to describe the use of antibiotics and post-operative infections.

**Methods:**

Patients with unilateral ZMC fractures between 2007-2018 and treatment with either open reduction and internal fixation (ORIF) or closed reduction (CR) were identified from medical records and invited to follow-ups between 2018-2020. Patients were examined, photographed, and completed a questionnaire. A review panel of 3 experienced surgeons evaluated photographs and computed tomography (CT) scans pre- and post-surgery.

**Results:**

The study sample consisted of 136 patients (108 ORIF, 28 CR) with a median follow-up time of 76 months. Patient satisfaction of surgical outcome was high (97.8%), with no significant differences in relation to the use of internal fixation, number of fixation points, or OF reconstruction. Dissatisfaction was primarily related to hypoesthesia. On post-operative CT scans, malar asymmetry was more often predicted in patients with 1-point fixations. On questionnaires and photographs, malar asymmetry was more common in patients with 3-point fixations. Prophylactic antibiotics had no effect on the rate of post-operative infections.

**Conclusions:**

Patient satisfaction was not influenced by internal fixation, number of fixation points, or OF reconstruction. Selected ZMC fractures can be treated with less invasive approaches. Caution should be observed when predicting long-term malar asymmetry on post-operative CT scans. The findings of this study highlight the importance of a rational and ethical use of surgery.

## Background

Zygomaticomaxillary complex (ZMC) fractures are commonly encountered at trauma centers around the world.^1^ Long-term complications include malar asymmetry, malposition of the globe, and sensory disturbances of the infraorbital nerve (ION). Rarely, diplopia and trismus are seen.^2,3^ The Zingg classification (1992)^4^ is commonly used to describe fractures that are incomplete (A), tetrapod (B), or multi-fragmented (C). A more recent modification by van Hout (2016)^5^ further defines incomplete fractures as those where at least 1 of the zygomatic pillars remains intact. Although many ZMC fractures can be treated conservatively, some will require surgery. This is typically done by closed reduction (CR) or open reduction and internal fixation (ORIF), where fracture lines are exposed and hardware, often plates and screws, are placed.^3^

Surgeons treat ZMC fractures in different ways and there is no consensus on when and how to perform a surgical reconstruction.^6^ A 3-point fixation at the zygomaticomaxillary buttress (ZMB), infraorbital rim (IOR), and frontozygomatic suture (FZ) has long been considered the best option and is still routinely used. Nevertheless, many fractures can be reconstructed with less invasive approaches, using fewer incisions and points of fixation.^3,4,7^

Several publications have described the number and location of fixation points required to achieve an acceptable reconstruction: 3-point fixations have often been compared to 1- and 2-point fixations, or no internal fixation at all.^8-10^ The results of these studies have been difficult to validate, and no consensus has been reached, partly due the use of different definitions and methods of selection, treatment, and evaluation.^10^ Similarly, the orbital floor (OF) is inconsistently reconstructed. Some surgeons routinely explore the OF after reducing the fractured zygoma, while others are selective and base their decision on the pre-operative computed tomography (CT) scan.^11-13^

The advantages of invasive surgery have been questioned, especially as many ZMC fractures only result in cosmetic sequelae.^6^ It is argued that multiple incisions, with higher risks of post-operative infections and scarring, should be weighed against the purpose of performing a surgical reconstruction. The perspectives of patients are important, but often overlooked.^2,6,10^

We have previously published a long-term follow-up of 180 patients with ZMC fractures.^2^ In this study, patients with both surgical and non-surgical treatments completed a questionnaire and underwent clinical examinations. Their CT scans (at diagnosis and immediately after surgery) and photographs (at long-term follow-up) were evaluated by a review panel of experienced surgeons. In summary, overall patient satisfaction of facial appearance was high without a significant difference between the treatment groups, although patients and surgeons perceived long-term functional sequelae differently. Furthermore, surgeon prediction of long-term malar asymmetry on CT scans neither correlated with their own assessments of photographs, nor with outcomes from patient questionnaires.

The present study describes patient perspectives in relation to the surgical treatment of ZMC fractures and comprises only a portion of the previously published cohort. The primary objective was to evaluate the long-term patient satisfaction of surgical outcome in relation to the use of internal fixation (CR/ORIF), number of fixation points, and OF reconstruction. Secondary objectives included an evaluation of the use of antibiotics and rates of post-operative infections.

## Materials and Methods

This retrospective study was conducted at the Karolinska University Hospital (KUH) in Stockholm, Sweden. A detailed description of materials and methods has been provided in our previous study,^2^ with a summary given below.

Adult patients treated for unilateral ZMC fractures between 2007-2018 were identified from hospital medical records and invited to clinical follow-up examinations between 2018-2020. Fractures were classified according to van Hout^5^ and were labeled type A (only 1 intact ZMC pillar), type B (displacement of all 4 ZMC pillars), or type C (multi-fragmented fractures). Patients were excluded if they had concurrent midfacial fractures, unretrievable pre-operative CT scans, incomplete contact information, language difficulties (not speaking Swedish or English), or significant co-morbidities preventing study participation. Treatment consisted of surgery with either CR or ORIF, using the *MatrixMIDFACE Plating System* (DePuy Synthes). No technical aids were used, such as virtual planning, intra-operative CT, and navigation systems. The use of prophylactic antibiotics and early post-operative follow-up were left to the discretion of the responsible surgeon.

During the follow-up visits, each patient underwent a clinical examination and was asked to complete a questionnaire on self-perceived functional and cosmetic sequelae. Photographs were taken from the frontal, basal, cephalic, oblique, and lateral angles. Pre-operative CT scans at the time of diagnosis and available post-operative CT scans were rendered into 3-dimensional (3D) models and saved in the same 5 angles as the patient photographs.

A review panel of 3 senior surgeons, with ongoing involvement in treating maxillofacial fractures, was formed. The panel was asked to evaluate pre- and post-operative CT scans, and to assess facial features on the photographs obtained during follow-up. No measurements were provided, and panel outcome was decided by majority vote.

### Clinical Examination

Patients were clinically examined for diplopia (present/absent). They were asked to fix their gaze on the movements of the examiner’s finger (in an H-shaped pattern) and report if they experienced double vision in any direction.

### Questionnaire

Patients were asked if they were satisfied with the surgical outcome (yes/no), and if they experienced malar asymmetry (yes/no), diplopia (yes/no), or sensory disturbances on the injured side of the face (yes/no). A space was also provided for additional comments.

### Photographs

The review panel evaluated the presence of malar asymmetry (yes/no) and orbital stigmas: enophthalmos (yes/no), hypoglobus (yes/no), and superior sulcus deformity (yes/no).

### CT Scans

On both pre- and post-operative CT scans, the review panel evaluated the degree of zygoma displacement (none/mild/severe) and the presence of significant OF displacement (absent/present). On post-operative scans, the panel also predicted whether the patient would suffer from future malar asymmetry (yes/no).

The degree of zygoma displacement was left to the discretion of the panel members and no instructions were provided apart from a classification of severity. Significant OF displacement was defined as herniation of orbital contents medial to the infraorbital nerve (ION), as seen on a pre-selected coronal image slice best deemed for such purpose. Throughout the study, malar asymmetry was defined as the injured side of the face or cheek being visibly sunken.

### Data Extraction

From the medical records, data was extracted on patient age, gender, dates of trauma and surgery, type of surgery, number and sites of internal fixation, number of and reasons for revisional surgery, use of prophylactic antibiotics, and post-operative infections. Data on post-operative infections was included from all documented cases within 30 days of surgery.

### Statistical Analysis

Statistical analysis was performed using IBM SPSS Statistics for Windows version 28 (IBM Corp., Armonk, NY, USA). Descriptive data was presented as total number (n), percentage of group (%), median, and interquartile range (IQR). Comparisons of groups were performed using Mann-Whitney U test for continuous data and Pearson’s chi-square test or Fischer’s exact test (when suitable) for dichotomous data. Statistical significance was set at the level of *P* < 0.05.

### Ethical Permission

This study was conducted in adherence to the Declaration of Helsinki and was approved by the regional ethical review authority (Etikprövningsnämnden) in Stockholm, Sweden (2018/302-31). All patients submitted an informed written consent for participation.

## Results

A total of 532 patients were invited to the follow-up, of which 180 patients (33.8%) attended. No significant differences were noted in age (*P* = 0.235) or gender (*P* = 0.916). Surgery was performed on 137 patients, of which 1 patient was excluded as treatment consisted of open reduction without fixation. The remaining 136 patients constituted the study sample (Table 1).

**Table 1.** Basic Data and Long-Term Patient Satisfaction Sorted by ORIF, CR, and the Total Study Sample. Findings are Expressed as Total Numbers and % (of Group), Except for Age at Trauma (Median Years, IQR) and Follow-Up Time (Median Months, IQR).

	All Patients (n = 136)	ORIF (n = 108)	CR (n = 28)	*P*-value
Male gender	114 (83.8)	91 (84.3)	23 (82.1)	0.777
Age at trauma	41.0 (28.0-52.5)	39.5 (27.0-53.5)	42.5 (28.5-50.0)	0.671
Fracture classification				
A	31 (22.8)	20 (18.5)	11 (39.3)	**0.020**
B	77 (56.6)	64 (59.3)	13 (46.4)	0.222
C	28 (20.6)	24 (22.2)	4 (14.3)	0.355
Follow-up time	76.0 (38.0-104.0)	69.0 (37.5-95.0)	96.5 (72.0-117.5)	**0.008**
Prophylactic antibiotics	77 (56.6)	76 (70.4)	1 (3.6)	**< 0.001**
Post-operative infection	22 (16.2)	22 (20.4)	0 (NA)	**0.007**
More than 1 surgery performed	7 (5.1)	7 (6.4)	0 (NA)	0.344
Satisfied patients	133 (97.8)	105 (97.2)	28 (100.0)	1.000

ORIF = open reduction and internal fixation; CR = closed reduction; IQR = interquartile range; NA = not applicable.

*Note*. Significance set at 5% (*P* < 0.05) and significant values marked in bold.

The median follow-up time was 76.0 months (38.0 - 104.0) after surgery. ORIF was performed on 108 patients (79.4%) and CR on 28 patients (20.6%). No significant differences were found in gender (*P* = 0.777) or age at trauma (*P* = 0.671). Long-term patient satisfaction of the surgical outcome was high (n = 133, 97.8%), without significant differences between treatment groups (*P* = 1.000) or fracture types (*P* = 0.649).

Patients treated with CR had a longer follow-up than patients with ORIF (96.5 vs 69.0 months, *P* = 0.008). CR was performed on 19 patients using a cutaneous traction hook around the zygomatic body and on 8 patients by introducing an elevator through a temporal fascia incision (Gillies approach). On 1 patient, both methods were combined to achieve reduction. CR was more often used for type A fractures (39.3% vs 18.5%, *P* = 0.020), and ORIF for type C fractures (22.2% vs 14,3%, *P* = 0.355). Out of 31 patients without a post-operative CT scan, 21 patients were treated with CR (67.7%, *P* < 0.001).

Prophylactic antibiotics were given to 77 patients (56.6%), and 76 of them were treated with ORIF (98.7%, *P* < 0.001). Among patients with ORIF, 70.3% were given prophylactics antibiotics. It was always administered during surgery as a single intravenous dose. In 49 cases (63.6%), antibiotics consisted of a combination of Penicillin G 3 g and Cloxacillin 2 g and in 23 cases (29.9%) of only Cloxacillin 2 g. In 5 cases (6.5%) other or unknown antibiotics were given. An extended oral course was continued after discharge for 2 patients (2.5%).

Post-operative infections within 30 days were seen in 22 patients, all of them treated with ORIF and a gingivo-buccal incision (16.2% of the study sample, 20.4% of patients with ORIF). Out of these, 15 patients (68.2%) were treated with prophylactic antibiotics. Most infections were successfully treated in an out-patient setting, although a second admission was required for 3 patients: 1 for abscess formation at the site of a submental intubation (surgically drained), 1 for spontaneous drainage of pus through a gingivo-buccal incision (conservative management), and 1 for abscess formation at the site of a lower eyelid incision (surgically drained).

In 129 patients (94.8%), a single surgical intervention was sufficient to reconstruct the ZMC fracture in a satisfactory manner. Early revisional surgery within 14 days was required for 4 patients due to insufficient reduction of the fracture (2 patients), poorly placed orbital floor plate, or insufficient intra-orbital fixation. Late secondary surgery was performed on 2 patients due to a poorly placed orbital floor plate (18 months) or cosmetic correction of disfiguring scar (24 months). One patient required 2 additional surgical procedures under general anesthesia for abscess drainage (11 days) and subsequent extraction of the internal fixation (45 days).

### Fixation Points

Fixation points are summarized in Table 2 and outcomes of review panel and patient questionnaire in Table 3. There were no significant differences in patient satisfaction in relation to the number of fixation points (*P* = 0.443). Out of 19 patients treated with 1-point fixations, 15 patients (78.9%) were plated at ZMB, 2 patients (10.5%) at FZ, and 2 patients (10.5%) at IOR. Out of 31 patients treated with 2-point fixations, 21 patients (67.7%) were plated at ZMB and IOR, 8 patients (25.5%) at ZMB and FZ, and 2 patients (6.5%) at FZ and IOR. A 3-point fixation at ZMB, IOR and FZ was given to 55 patients and a 4-point fixation, with an additional plate at the zygomatic arch (ZA), to 3 patients.

**Table 2.** Number of Fixation Points and Long-Term Patient Satisfaction of Each Group Sorted by Fracture type. Findings of treatment type are expressed as total numbers and % (of fracture type). Findings of patient satisfaction are marked in italic and expressed as total numbers and % (of treatment type).

	Type A (n = 31)	Type B (n = 77)	Type C (n = 28)	All Patients (n = 136)
No fixation	11 (35.5)	13 (16.9)	4 (14.3)	28 (20.6)
CR	11 (35.5)	13 (16.9)	4 (14.3)	28 (20.6)
Satisfied patients	*11* (*100.0)*	*13* (*100.0)*	*4* (*100.0)*	*28 (100.0)*
1-Point fixation	5 (16.1)	12 (15.6)	2 (7.1)	19 (14.0)
ZMB	5 (16.1)	10 (13.0)	0 (NA)	15 (11.0)
IOR	0 (NA)	2 (2.6)	0 (NA)	2 (1.5)
FZ	0 (NA)	0 (NA)	2 (7.1)	2 (1.5)
Satisfied patients	*5* (*100.0)*	*10* (*83.3)*	*2* (*100.0)*	*17 (89.5)*
2-Point fixation	8 (25.8)	18 (23.4)	5 (17.9)	31 (22.8)
ZMB+IOR	6 (19.4)	10 (13.0)	5 (17.9)	21 (15.4)
ZMB+FZ	1 (3.2)	7 (9.1)	0 (NA)	8 (5.9)
IOR+FZ	1 (3.2)	1 (1.3)	0 (NA)	2 (1.5)
Satisfied patients	*8* (*100.0)*	*18* (*100.0)*	*5* (*100.0)*	*31 (100.0)*
3-Point fixation	7 (22.6)	34 (44.2)	14 (50.0)	55 (40.4)
ZMB + IOR + FZ	7 (22.6)	34 (44.2)	14 (50.0)	55 (40.4)
Satisfied patients	*7* (*100.0)*	*33* (*97.1)*	*14* (*100.0)*	*54 (98.2)*
4-Point fixation	0 (NA)	0 (NA)	3 (10.7)	3 (2.2)
ZMB + IOR + FZ + ZA	0 (NA)	0 (NA)	3 (10.7)	3 (2.2)
Satisfied patients	*0* (*NA)*	*0* (*NA)*	*3* (*100.0)*	*3 (100.0)*

CR = closed reduction; ORIF = open reduction and internal fixation; ZMB = zygomaticomaxillary buttress; IOR = infraorbital rim; FZ = frontozygomatic suture; ZA = zygomatic arch; NA = not applicable.

**Table 3.** Zygoma Displacement and Malar Asymmetry, Sorted by Number of Fixation Points. Malar Asymmetry was Reported by Patients (Using a Questionnaire) and Evaluated by the Review Panel (on Photographs Taken at Follow-Up and Post-operative CT Scans). Findings are Expressed as Total Numbers and % (of Group).

	All ORIF (n = 108)	1-Point Fixation (n = 19)	2-Point Fixation (n = 31)	3-Point Fixation (n = 55)	*P*-value
Zygoma displacement on pre-op CT scans					
No displacement	0 (NA)	0 (NA)	0 (NA)	0 (NA)	NA
Mild displacement	55 (50.9)	16 (84.2)	19 (61.3)	20 (36.4)	**< 0.001**
Severe displacement	53 (49.1)	3 (15.8)	12 (38.7)	35 (63.6)	**< 0.001**
Zygoma displacement on post-op CT scans					
No displacement	34 (31.5)	4 (21.1)	9 (29.0)	21 (38.2)	0.446
Mild displacement	62 (57.5)	11 (57.9)	19 (61.3)	29 (52.7)	0.525
Severe displacement	2 (1.9)	1 (5.3)	0 (NA)	1 (1.8)	0.395
Malar asymmetry					
Questionnaire	14 (13.0)	1 (5.3)	4 (12.9)	8 (14.5)	0.668
Photographs	29 (26.9)	2 (10.5)	5 (16.1)	21 (38.2)	**0.018**
Post-operative CT scans	21 (19.4)	6 (31.6)	5 (16.1)	9 (16.4)	0.345
Post-operative CT scan not performed	10 (9.3)	3 (15.8)	3 (9.7)	4 (7.3)	0.489

ORIF = open reduction and internal fixation; CT = computed tomography; NA = not applicable.

*Note*. Significance set at 5% (*P* < 0.05) and significant values marked in bold.

All patients had some degree of zygoma displacement on pre-operative CT scans. Cases with mild displacement were favored for 1-point fixations (*P* < 0.001), and cases with severe displacement for 3-point fixations (*P* < 0.001). Post-operative CT scans revealed that more than half of the patients (57.5%) with ORIF suffered a mild, residual post-operative displacement of the zygoma. Patients with 3-point fixations had the highest rate of no residual displacement (38.2%, *P* = 0.446).

Malar asymmetry was more often reported by patients (*P* = 0.668) and detected on photographs (*P* = 0.018) by the review panel as the number of fixation points increased and was highest among patients with 3-point fixations. Conversely, when the review panel evaluated post-operative CT scans, malar asymmetry was more often predicted among patients with 1-point fixations (*P* = 0.345).

In the total study sample, 85 patients reported post-operative sensory disturbances: 13 patients treated with CR (46.4%), 10 patients with 1-point fixations (52.6%), 19 patients with 2-point fixations (61.3%), 41 patients with 3-point fixations (74.5%), and 2 patients with 4-point fixations (66.7%).

The 3 patients who stated dissatisfaction with the surgical outcome were all treated with ORIF, and 2 of them spontaneously stated hypoesthesia as a significant reason:

Patient 1. A type B fracture treated with a 1-point fixation at ZMB with severe post-operative displacement and predicted malar asymmetry on the post-operative CT scan. Antibiotics were prescribed after 4 days for fever and general swelling of the cheek. The patient expressed dissatisfaction at follow-up after 78 months due to hypoesthesia.
Patient 2. A type B fracture treated with a 3-point fixation with no displacement or predicted malar asymmetry on the post-operative CT scan. There was no post-operative infection. The patient expressed dissatisfaction at follow-up after 81 months due to hypoesthesia and protrusion of the injured cheek.
Patient 3. A type B fracture treated with a 1-point fixation at ZMB with no displacement or predicted malar asymmetry on the post-operative CT scan. There was no post-operative infection. No specific comments were provided regarding the reason for dissatisfaction at follow-up after 23 months.

### Orbital Floor Reconstruction

OF reconstruction (Table 4) was performed on 11 out of 108 patients treated with ORIF (10.1%), although 34 patients (31.5%) were noted to have a pre-operative displacement of the OF. There were no significant differences in patient satisfaction with regards to OF reconstruction (*P* = 1.000) or post-operative OF displacement (*P* = 0.202).

**Table 4.** Significant orbital floor displacement, Orbital Stigmas, and Outcomes of Patient Questionnaire and Clinical Examinations, Sorted by orbital floor reconstruction. Findings are Expressed as Total Numbers and % (of Group).

	All ORIF (n = 108)	OF Reconstruction (n = 11)	No OF Reconstruction (n = 97)	*P*-value
Pre-operative CT scans				
No OF displacement	74 (68.5)	3 (27.3)	71 (73.2)	**0.004**
OF displacement	34 (31.5)	8 (72.8)	26 (26.8)	
Post-operative CT scans				
No OF displacement	81 (75.0)	10 (90.9)	71 (73.2)	0.202
OF displacement	17 (15.7)	0 (NA)	17 (17.5)	
Post-operative CT scan not performed	10 (9.3)	1 (9.1)	9 (9.3)	1.000
Photograph evaluation				
Any orbital stigma	19 (17.6)	4 (36.4)	15 (15.4)	0.101
Enophthalmos	13 (12.0)	1 (9.1)	12 (12.4)	1.000
Hypoglobus	5 (4.6)	0 (NA)	5 (5.2)	1.000
Superior sulcus deformity	12 (11.1)	4 (36.4)	8 (8.2)	**0.019**
Clinical examination				
Diplopia	1 (0.9)	0 (NA)	1 (1.0)	1.000
Questionnaire				
Diplopia	4 (3.7)	2 (18.2)	2 (2.1)	0.051
Satisfied patients	105 (97.2)	11 (100.0)	94 (96.9)	1.000

ORIF = open reduction and internal fixation; OF = orbital floor; CT = computed tomography; NA = not applicable.

*Note*. Significance set at 5% (*P* < 0.05) and significant values marked in bold.

On photographs taken at follow-up, the review panel detected orbital stigmas–mostly enophthalmos–in 19 patients (17.6%). Among patients with OF reconstruction, 4 cases of superior sulcus deformity were found (36.4% vs 8.2%, *P* = 0.019), and it was noted that 3 of them did not have any other orbital stigma.

## Discussion

The main finding of this study was the high long-term patient satisfaction of the surgical outcome following reconstruction of ZMC fractures. There were no significant differences in relation to the use of internal fixation or OF reconstruction. Although 3-point fixations appeared to result in better post-operative results on CT scans, the number of fixation points did not yield a significant difference in long-term patient satisfaction. These findings suggest that patients with ZMC fractures are generally satisfied with the outcome several years after surgery. Not all fractures require multiple fixation points or an optimal anatomical reconstruction on the post-operative CT scan. Less invasive treatments, when rationally selected, also have the potential of resulting in high patient satisfaction.

The goal of reconstructing ZMC fractures is to achieve anatomical reduction and stability over time, with surgeons often using radiological outcomes to measure success.^4,10^ Historically, a buttress-based approach has been used to address the number and location of points requiring fixation, and a 3-point fixation at ZMB, IOR and FZ has long been considered robust and reliable.^3^ Nevertheless, over time, it has emerged that many fractures can be successfully reconstructed with less fixation points. For example, a previous publication from our institution showed that CR is suitable for fractures with little to moderate displacement, although it may prove inadequate for fractures where displacement is more severe.^14^

Facial trauma surgeons are now increasingly concerned with treatment sequelae, especially scar formations.^3^ By using an incision-based approach, treatments are individualized; the aim is to use only as many incisions as necessary to achieve the purpose of a reconstruction. During surgery, continual evaluation is made to determine whether additional incisions are required.^6^

### Patient Satisfaction

Our study revealed a high long-term patient satisfaction with the surgical outcome, consistent with previous studies describing a high long-term health related quality of life (HRQoL) among patients with ZMC fractures.^15,16^ The number of fixation points used for reconstruction did not significantly influence patient satisfaction, aligning with Nasr et al (2017)^17^ but differing from Kim et al (2011),^18^ who noted that patients with 2-point fixations were less satisfied compared to those with 1-point fixations. They associated dissatisfaction with post-operative scars, while long-term dissatisfaction in our study was mainly attributed to hypoesthesia.

Both Kaukola et al (2017)^15^ and Kurita et al (2010)^19^ identified sensory disturbances as the most common complication to ZMC fractures. In our study, more than half of the patient reported sensory disturbances at follow-up. As surgery became more invasive (i.e. more use of incisions and internal fixation), sensory disturbances became more common. It should, however, be noted that invasive treatments were primarily given to displaced fractures. Unfortunately, our documentation of sensory disturbances before surgery was inconsistent and could not be described.

Although the 3 dissatisfied patients were all treated with ORIF, there was no significant difference in patient satisfaction in relation to the use of internal fixation, aligning with the findings of Alshalah et al (2023).^20^ The median follow-up time for patients treated with CR was significantly longer than for patients treated with ORIF. This reflects a change in practice, as CR was during the initial study phase more commonly used in treating ZMC fractures.^14^ In addition, the routine of consistently performing post-operative CT scans was during the study period gradually introduced, explaining why more than half of the patients with CR did not perform a post-operative CT scan.

### Fixation Points

Many previous studies have tried to describe an ideal number and combination of fixation points. A systematic review by Gadkari et al (2019)^8^ found that 5 of 8 studies were in favor of 3-point fixations compared to 2-point fixations, concluding that a 3-point fixation could be used as a standard treatment for ZMC fractures. Another systematic review by Jazayeri et al (2019)^9^ included 13 studies, of which a meta-analysis was conducted on 2 randomized controlled trials. The findings indicated that 3-point fixations led to enhanced fracture stability and reduced the likelihood of globe malposition compared to 2-point fixations. A third systematic review by Raghoebar et al (2021)^10^ included 17 studies and highlighted the difficulty in drawing reliable conclusions regarding no fixation, 1-point fixation, and multiple points of fixation, partly due to different methods and outcome measures used among studies. All 3 systematic reviews stressed the poor quality of evidence.

Several pitfalls exist when comparing fixation points. Less fixation can be assumed to result in biomechanical instability, although the quality of reduction–before the application of fixation–is rarely described.^7^ An advantage of using multiple incisions can be increased visibility to evaluate the reduction, rather than the opportunity to place additional plates. Moreover, fracture stability over time can only be assessed by comparing immediate post-operative CT scans to additional scans taken at a later stage, a method used only by few studies.^10^

As a result of selection bias, there were significant differences in our choice of treatment depending on fracture type and the amount of displacement seen on the pre-operative CT scan. Patients with mild displacement were favored for less invasive surgery (i.e. 1-point fixations), and patients with severe displacement for more invasive surgery (i.e. 3-point fixations). Still, it could be noted among patients with ORIF, that more than 20% of type A fractures received 3-point fixations and almost 40% of type C fractures received less than 3 fixation points. An inconsistent use of fixation points was expected, as treatments were based on surgeon preference rather than specific protocols.

### Evaluating Surgical Treatment

In our previous study, we noted the absence of a significant correlation between long-term malar asymmetry predicted on CT scans and long-term follow-up findings on photographs and outcomes of patient questionnaires.^2^ The unreliability of predicting long-term malar asymmetry on CT scans was further demonstrated in our present study. Despite surgical outcomes being deemed satisfactory at the time of treatment, the review panel noted on post-operative scans that more than half of the patients had some degree of residual displacement.

Furthermore, patients with 3-point fixations had the highest rates of malar asymmetry on questionnaires and photographs. Surprisingly, evaluation of post-operative CT scans revealed the opposite, with the highest rate of malar asymmetry predicted among patients with 1-point fixations. Given the limitations of a small sample size and the use of photographs to evaluate malar asymmetry, one explanation to this contrast may be a long-term thinning or atrophy of the soft tissues, and the illusion of a sunken cheek, resulting from invasive surgery. Further studies are needed to validate this finding, especially as patients with ZMC fractures are usually only followed for a limited time after surgery.

Taken together, our results confirm the robustness and adequacy of the 3-point fixation, particularly when evaluating post-operative outcomes on CT scans. However, when assuming a broader perspective that includes patient satisfaction, it appears that many ZMC fractures can also be successfully treated with less invasive approaches.

### Prophylactic Antibiotics and Infections

Prophylactic antibiotics are inconsistently used in the management of maxillofacial fractures, although some general principles have previously been described. Fractures involving the mandible and frontal sinuses pose increased risks of infection compared to those involving the midface.^21,22^ Infections following ZMC fractures are traced to the surgical intervention rather than the fracture itself, in particular when surgeons use intra-oral approaches.^22,23^ Extended post-operative antibiotic courses provide no advantage compared to antibiotics given at the time of surgery.^24,25^ Given these reports, the need and adequate use of prophylactic antibiotics when treating ZMC fractures is still to be further explored.^26^

In our study, both prophylactic antibiotics and post-operative infections were primarily related to patients treated with ORIF, which was expected given the increased risks of infections following a gingivo-buccal incision. Among these patients, prophylactic antibiotics appeared to have no effect on the occurrence of a post-operative infection. Overall, our infection rates were higher than previously published reports, where well-defined and more strict definitions of post-operative infections have been used.^23-25^ Our method of following patients immediately after surgery was inconsistent, as clinical assessments were performed differently and at times by physicians other than the main surgeon. Consequently, all cases with post-operative infection on record were included in the study.

### Orbital Floor Reconstruction

Criteria for surgical intervention in isolated blow-out fractures (BOF) are often based on the pre-operative CT scan, where the amount of OF displacement and herniation of orbital contents decide whether a reconstruction is required to avoid orbital stigma.^27,28^ In ZMC fractures, some have argued that the pre-operative CT scan is unreliable, as reduction of the zygoma can alter the shape of the OF and cause iatrogenic indications for OF reconstruction (though the resultant effect remains a topic of debate). Therefore, while some propose the use of pre-operative CT scans to decide whether OF reconstruction is required, others recommend routine exploration of the OF during surgery.^11,13,29^ There is to date no consensus on how to approach OF reconstruction in ZMC fractures.

Most ZMC fractures will cause a minimal disruption of the OF lateral to the infraorbital canal.^28^ In the absence of a consensus on when OF reconstruction should be performed, we defined significant OF displacement as cases where the fracture and orbital herniation extended medial to the ION. Using this definition, a significant difference was seen in treatment depending on whether the pre-operative CT scan showed signs of significant OF displacement. However, it could also be noted that less than a fourth of patients with significant OF displacement were reconstructed and 3 patients without significant displacement received an orbital floor plate. Inadequate or unsuccessful OF repair necessitated revisional surgery in 3 cases, and negative OF explorations were documented in several instances. These examples show ambiguity with regards to when the OF should be reconstructed.

It was therefore expected that cases of enophthalmos and hypoglobus were more often seen in patients without OF reconstruction. However, it was surprising that 3 out of the 11 patients with OF reconstruction had isolated superior sulcus deformity, without any other orbital stigma. A deep sulcus has previously been described in patients with conservatively treated BOF, suggestive of it being a trauma-related stigma rather than surgically induced.^30^

In summary, it can be said that the question of when and how to operate ZMC fractures cannot easily be answered. These patients constitute a diverse group, with fractures that are often difficult to categorize and study. The literature suggests that due attention is increasingly being given to the underlying purposes of surgery, while patient perspectives are being considered; the success or failure of a reconstruction is not merely judged by the appearance of the repaired fracture on a CT scan.

Many patients, especially after a recent physical and psychological trauma, and grappled by pain, swelling, and numbness, overestimate the significance of their ZMC fracture and the often minimal cosmetic results. Some erroneously believe that an invasive surgical intervention is necessary for optimal recovery. Our study’s main finding of a high long-term patient satisfaction with the surgical outcome, regardless of whether invasive surgery was used, is in an important one, and should serve as a reminder of a rational and ethical approach to surgical decision-making.

### Limitations

It is important to interpret our results carefully considering the study’s limitations and the complexity of managing ZMC fractures. The retrospective nature of the study resulted in a significant dropout rate and a limitation of the number of study participants, particularly relevant when drawing statistical conclusions. Patients sustained isolated ZMC fractures without concurrent midfacial fractures and treatments were not guided by computer-aided technology or intra-operative CT. Furthermore, we used a non-validated patient questionnaire and assessed OF displacement based on a single coronal image slice rather than complete CT scans. Lastly, our evaluation of orbital stigmas and malar asymmetry relied on patient photographs rather than comprehensive clinical examinations. Further research with larger sample sizes and controlled study designs may provide more robust evidence regarding the comparative effectiveness of different treatment approaches.

## Conclusion

Most patients with zygomaticomaxillary complex fractures are satisfied with the surgical outcome several years after treatment. No significant differences in long-term patient satisfaction have been observed in relation to the use of internal fixation, number of fixation points, or reconstruction of the orbital floor. Prediction of long-term malar asymmetry should be made with caution, especially if only based on post-operative computed tomography scans. Future studies are needed to establish clear indications for zygomaticomaxillary complex fracture reconstruction and concomitant orbital floor repair.
